# 

**Published:** 1809-03

**Authors:** 


### NEW MEDICAL PUBLICATIONS.

Anatomico-Chirurgical Views of the Nose, Mouth, Larynx, and Fauces; with appropriate Explanations and References. By John James Watt, Surgeon. Together with an additional Anatomical Description of the Parts, by Mr. W. Lawrence, Demonstrator of Anatomy at St. Bartholomew's Hospital. The engravings executed by Mr. Hopwood, from original drawings by Mr. T. Baxter. Folio, price 1l. 11s. 6d. plain, or 2l. 2s. coloured.

The London Medical Review, Vol. I. 12s. 6d.

A Letter to John Haygarth, M. D. from Colin Chisholm, M. D. Author of an Essay on the Pestilential Fever, exhibiting further Evidence of the infectious Nature of this fatal Distemper in Grenada during 1793, 4, 5, and 6; and in the United States of America, from 1793 to 1805; in order to correct the pernicious Doctrine promulgated by Dr. Edward Miller and other American Physicians, relative to this destructive Pestilence. 8vo. 6s.

Observations on the Proceedings and Report of the Special Medical Board, appointed to examine the State of the Army Depot Hospital in the Isle of Wight. By Thomas Keate, Esq. Surgeon-general to the Forces. 3s. 6d.

Observations on the Management of the Insane, and particularly on the Agency and Importance of humane and kind Treatment in effecting their Cure. By Thomas Arnold, M. D. 3s.

An Essay on Warm and Vapor Baths, with Hints for a new Mode of applying Heat and Cold, for the Cure of Disease and the Preservation of Health. By Edward Kentish, M. D. 8vo. 4s. 6d.

---

### To CORRESPONDENTS.

Dr. Mossman must have misunderstood us very much, if he conceived we had any objection to our Correspondents supporting their opinions by quotations, however numerous. If he will permit us to omit a few passages in the beginning of his letter, which we believe a re-perusal would induce him to be at least indifferent about, the rest shall be inserted with *all his quotations*. For his communication we find ourselves indebted, but we doubt not Dr. M's good intentions would induce him anxiously to avoid every thing which might protract the severity of a controversy.

We have received a *Reply to Joannes versus Jacobus*, in our last. The most candid notice we can take of it is to suppose, that the latter, living at a great distance from the metropolis, or from any metropolis, is ignorant of what is going on in those places; he would otherwise have known that potash is decomposed by iron, in consequence of a double affinity, as Mr. Davy has taught us at the Royal Society, and that it cannot be decompos- by charcoal only. Yet Jacobus seems, in other respects, to know more than those who have better means of information. In his letter, which bears the post mark of more than 100 miles from London, he tells us the arsenic acid *is said* to be negative. We are not told by whom this *is said*. If Jacobus, who is anonymous, has only heard it of somebody whom he does not wish to name, he surely cannot expect us to reason on such authority. If he means to say that *it is said* by Mr. Davy, we are obliged to add that we never heard it from that gentleman, nor, on enquiry, can we learn that he has communicated any experiments on arsenic acid. We should not have taken so much notice of this communication, were it not that the writer seems to think his opponent has viewed things through a prejudiced medium. By these few remarks he will perceive that his paper is not rejected without having met with proper attention.

We have received several remarks on the New Vaccine Institution, but till we are better acquainted with its constitution and proceedings we forbear to insert any of them.

Communications are received from Dr. Pole, Dr. Roberton, Mr. Hall, and several anonymous.

